# CD34-positive cells and their subpopulations characterized by flow cytometry analyses on the bone marrow of healthy allogenic donors

**DOI:** 10.1590/S1516-31802009000100004

**Published:** 2009-05-11

**Authors:** Jerusa Martins Carvalho, Marlon Knabben de Souza, Valéria Buccheri, Cláudia Viviane Rubens, José Kerbauy, José Salvador Rodrigues de Oliveira

**Affiliations:** 1 BSc. Postgraduate student, Division of Hematology and Transfusion Medicine, Hospital São Paulo, Universidade Federal de São Paulo - Escola Paulista de Medicina (Unifesp-EPM), São Paulo, Brazil.; 2 MD. Postgraduate student, Division of Hematology and Transfusion Medicine, Hospital São Paulo, Universidade Federal de São Paulo - Escola Paulista de Medicina (Unifesp-EPM), São Paulo, Brazil.; 3 MD, PhD. Associate professor, Division of Hematology, Department of Medicine, Universidade de São Paulo (USP) and Head of Laboratory of Cell Biology, Fundação Maria Cecília Couto Vidigal, São Paulo, São Paulo, Brazil.; 4 BSc. Biochemistry researcher in the Cell Biology Laboratory, Fundação Maria Cecília Couto Vidigal, São Paulo, São Paulo, Brazil.; 5 MD, PhD. Titular professor, Division of Hematology, Department of Medicine, Universidade Federal de São Paulo - Escola Paulista de Medicina (Unifesp-EPM), São Paulo, Brazil.; 6 MD, PhD. Associate professor, Division of Hematology, Department of Medicine, Universidade Federal de São Paulo - Escola Paulista de Medicina (Unifesp-EPM), São Paulo, Brazil.

**Keywords:** Hematopoietic stem cells, Immunophenotyping, Antigens, CD34, Hematopoiesis, Bone marrow transplantation, Células-tronco hematopoéticas, Imunofenotipagem, Antígeno CD34, Hematopoese, Transplante de medula óssea

## Abstract

**CONTEXT AND OBJECTIVE:**

Counting and separating hematopoietic stem cells from different sources has importance for research and clinical assays. Our aims here were to characterize and quantify hematopoietic cell populations in marrow donors and to evaluate CD34 expression and relate this to engraftment.

**DESIGN AND SETTING:**

Cross-sectional study on hematopoietic stem cell assays, using flow cytometry on donor bone marrow samples, for allogenic transplantation patients at two hospitals in São Paulo.

**METHODS:**

Immunophenotyping of marrow cells was performed in accordance with positive findings of CD34FITC, CD117PE, CD38PE, CD7FITC, CD33PE, CD10FITC, CD19PE, CD14FITC, CD13PE, CD11cPE, CD15FITIC, CD22PE, CD61FITC and CD56PE monoclonal antibodies in CD45PerCP+ cells, searching for differentiation and maturation regions. CD34+ sorting cells were analyzed for CD38 and CD117. Rh-123 retention was done before and after sorting. Antigen expression and CD34+ cells were correlated with engraftment.

**RESULTS:**

In region R1, 0.1% to 2.8% of cells were CD34+/CD45+ and 1.1%, CD34+/CD45-. The main coexpressions of CD45+ cells were CD38, CD22, CD19 and CD56 in R2 and CD33, CD11c, CD14, CD15 and CD61 in R3 and R4. After sorting, 2.2x10^6^ CD34+ cells were equivalent to 4.9% of total cells. Coexpression of CD34+/CD38+ and CD34+/CD117+ occurred in 94.9% and 82% of events, respectively. There was a positive relationship between CD34+ cells and engraftment. More than 80% of marrow cells expressed high Rh-123. CD34+ cell sorting showed that cells in regions of more differentiated lineages retained Rh-123 more intensively than in primitive lineage regions.

**CONCLUSION:**

We advocate that true stem cells are CD34+/CD45-/CD38-/low-Rh-123 accumulations.

## RESUMO

**CONTEXTO E OBJETIVO:**

A contagem e separação de células-tronco hematopoéticas de diferentes fontes tem importância para ensaios clínicos e pesquisa basica. Nosso objetivo foi caracterizar e quantificar as populacões de células hematopoéticas, bem como avaliar a expressão do antígeno CD34 em populações mais primitivas e correlacioná-las com a enxertia nos doadores de medula óssea para transplante alogênico.

**TIPO DE ESTUDO E LOCAL:**

Estudo transversal no qual a diferenciação e a seleção de células-tronco hematopoéticas foram realizadas em amostras de medula óssea de doadores de pacientes submetidos a transplante alogênico nos Hospitais São Paulo e Santa Marcelina, São Paulo, Brasil.

**MÉTODOS:**

Imunofenotipagem de células mononucleares de medula óssea foi feita na população de células CD45PerCP+ com os seguintes anticorpos: CD34FITC, CD117PE, CD38PE, CD7FITC, CD33PE, CD10FITC, CD19PE, CD14FITC, CD13PE, CD11cPE, CD15FITC, CD22PE, CD61FITC e CD56PE. Após a definição de regiões de células positivas ao CD34, estas células foram selecionadas e analisadas para a co-expressão do CD38 e CD117. Células mononucleares totais de medula óssea e aquelas obtidas após a seleção foram testadas para a retenção de Rh-123. O teste de Friedman e o coeficiente de Sperman foram utilizados para comparar as expressões e correlacionar a contagem de células CD34+ com a enxertia.

**RESULTADOS:**

Na região R1, 0,1% a 2,8% das células foram CD34+/CD45+, porém apenas 1,1% das células foram CD34+/CD45-. As principais co-expressões de células CD45+ foram CD38, CD22, CD19 e CD56 na região R2 e CD33, CD11c, CD14, CD15 e CD61 nas regiões R3 e R4. Após a seleção, a mediana de 2,2x106 células CD34+ foi equivalente a 4,9% do total mediano de células da medula óssea. Co-expressões de células CD34+/CD38+ e CD34+/CD117+ ocorreram em 94,95 e 82%, respectivamente. Houve relação positiva entre o número de células CD34+ infundidas e o dia da enxertia. Observamos que mais de 80% das células mononucleares de medula óssea retêm intensamente a Rh-123. Após a seleção, células localizadas em regiões de maior diferenciação, regiões R3 e R4, acumulam mais fortemente a Rh-123 do que células mais primitivas da região R1.

**CONCLUSÃO:**

Postulamos que a célula tronco hematopoética mais primitiva expressa o seguinte fenótipo: CD34+/CD45-/CD38-/Rh-123 de baixa retenção.

## INTRODUCTION

Quantification of CD34+ cells has an essential role in hematopoietic stem cell transplantation, whether autologous or allogenic, since the engraftment itself results from proliferation of the true hematopoietic stem cells. On the other hand, the progenitor cells expressing CD34 on the surface, which are already committed to some level of differentiation, sustain hematopoiesis only temporarily.^1^^,^^2^

The CD34 antigen is highly expressed in pluripotent cells, and its expression gradually reduces as the level of maturation of hematopoietic cell lineages increases, to the point of becoming completely absent in fully mature cells.^3^^,^^4^^,^^5^ By immunophenotyping CD34+ cells, it becomes evident that more than 95% of these cells are already committed to a particular lineage, since they also express lymphoid or myeloid-specific antigens. Prominent among these antigens is CD38, which is positively expressed in more than 90% of CD34+ cells. With regard to CD38 expression intensity, hematopoietic progenitors are classified as early (low CD38 expression) or differentiated progenitors (higher CD38 expression). A human B-cell/myeloid common progenitor expressing CD34+/CD38^low^/CD19+/CXCR4- has been identified.^6^

Other molecules may be useful in identifying marrow progenitors, especially CD117 (c-kit). This proto-oncogene codes for a protein receptor present in CD34+ cells. CD117 ligand (c-kit ligand), or stem cell factor, is produced by stromal cells. Achieving interaction between CD117 and its ligand is one of the necessary steps in the maturation process for marrow cells, together with interactions with other growth factors.^5^^,^^7^

B-lineage immature cells characteristically express CD34, HLA-DR and high-intensity CD10 (also known as CALLA), while B cells with greater differentiation express CD45, CD22, CD19, TdT and low-intensity CD10.^6^^,^^8^ Myeloid progenitors positively express CD34, CD33 and CD13 in more immature cells; and CD11b, CD11c and CD15 are expressed mainly in mature granulocytic cells. Monocytic cells express CD14, and CD61 is specific to cells committed to megakaryopoiesis^9^^,^^10^ The thrombopoietin receptor is now an important marker for separating granulocyte/monocyte from megakaryocyte/erythroid precursors, because of difficulty in distinguishing CD19-/CD34+/IL-3Ra^low^-/CD45RA-/TpoR- from CD19-/CD34+ /IL-3Ra^low^+/CD45RA-/TpoR+ based only on IL-Ra^low^ expression.^9^^,^^10^ Rhodamine-123 (Rh-123) is a useful tool for identifying hematopoietic cells that are more primitive. This substance accumulates in metabolically active cells. In hematopoietic cells, the number of mitochondria is small, reflecting their low metabolic rate, and this leads to low accumulation of Rh-123. On the other hand, cells with greater differentiation show a higher metabolic rate and accumulate more Rh-123.^11^

## OBJECTIVE

In this report, our objectives were the following: to use flow cytometry to characterize and quantify different populations of hematopoietic cells in healthy marrow donors; to evaluate CD34 expression and relate this to engraftment day; and to evaluate the pattern of CD34 expression in relation to Rh-123 retention and CD38 and CD117 coexpression in CD34+/CD45- cells.

## METHODS

Eleven marrow samples (10 ml) from healthy donors that were obtained for allogenic stem cell transplantation were analyzed. Among the marrow recipients, seven were diagnosed with chronic myeloid leukemia, one with multiple myeloma, one with severe aplastic anemia, one with lymphoblastic T-cell non-Hodgkin’s lymphoma and one with Philadelphia-positive acute lymphoblastic leukemia.

Mononuclear cells were separated by means of Ficoll-Hypaque gradient. Buffy coats were washed and resuspended in phosphate-buffered saline (PBS). After performing cell counts and evaluating cell viability, the cell samples were cryopreserved using 60% Roswell Park Memorial Institute (RPMI) medium, 20% fetal calf serum and 20% dimethyl sulfoxide solution (one part of cell suspension to one part of cryopreserved solution). Freezing was performed gradually until the temperature declined to 8 °C, -20 °C, -80 °C and -196 °C. Defrosting was performed using double boiling, followed by rinsing with RPMI. Finally, Buffy coats were resuspended in PBS.

For immunophenotyping, 50 µl of PBS-azide (pH 7.4) and the following fluorochrome-conjugated monoclonal antibodies were added to the PBS-marrow suspension: CD34FITC, CD117PE, CD38PE, CD7FITC, CD33PE, CD10FITC, CD10PE, CD14FITC, CD13PE, CD11cPE, CD15FITIC and CD56PE. These were diluted in accordance with the manufacturer’s specifications and their associations with CD45PerCP were tested. After a short incubation time, the cells were rinsed with 2 ml of PBS-azide and finally resuspended in 1% paraformaldehyde until the time of data acquisition.

The data acquisition and analysis were performed using Becton-Dickinson FACScalibur and CellQuest software. We established that the minimum number of events to be acquired prior to analysis needed to be 25,000. The positive control was CD45 and the negative control was *gamma-1 gamma-1*.

After defining the region of CD34 positivity, CD34-positive cells were sorted among ten of eleven samples, for further analysis regarding the expression of CD38 and CD117.

In the next step, the marrow mononuclear cell suspension (10^6^ cells/ml) was incubated for 20 minutes with a solution of PBS, fetal calf serum, water and Rh-123 (1 µg/ml). The cells were then washed with RPMI twice, resuspended in PBS/0.25% fetal calf serum and kept on ice, protected from light, until data acquisition. The analysis of Rh-123 accumulation, compared with CD34 positivity, was performed using Paint-a-gate (BD) software.

The Friedman test and Spearman coefficient were used to compare the median percentages of each specific CD expression, and to correlate the absolute number of CD34+ cells with the day of engraftment.

## RESULTS

**Figure 1.**
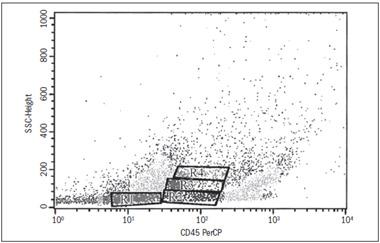
Immunophenotypic characterization of marrow stem cells. Distribution of marrow cells according to CD45 expression and complexity. Four regions can be identified (R1-R4).

Four regions were defined, according to the positivity pattern of the antigens studied (R1, R2, R3 and R4) (Figure 1). Considering that CD45 is almost completely absent in more primitive marrow cells and that its expression differs among lineage-committed progenitors (lymphoid, myeloid, megakaryocytic and natural killer cells), we chose to analyze its expression in relation to specific lineage determinants, labeled with different fluorochromes. We started by characterizing the CD34 and then identified the cells with greater differentiation that were already committed to any of the above mentioned cell lines, to express lineage-specific antigens.

**Figure 2.**
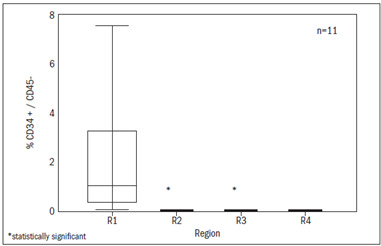
Immunophenotypic characterization of marrow stem cells. Hematopoietic stem cells (CD34+/CD45-) are significantly more concentrated in region 1. Samples from 11 patients were analyzed.

**Figure 3.**
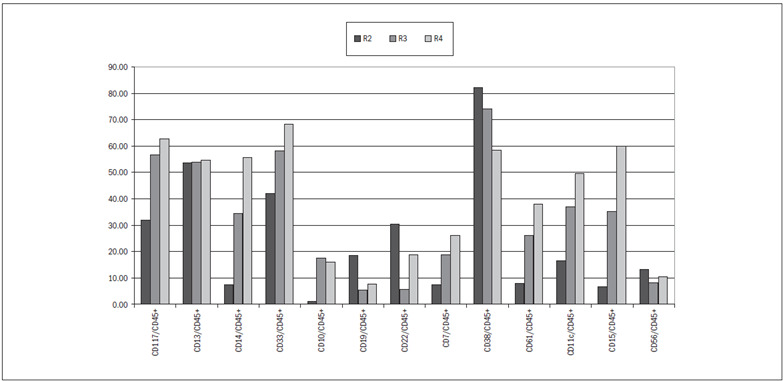
Immunophenotypic characterization of marrow stem cells. CD45+ cells are distributed predominantly in regions R2, R3 and R4 and they share markers of myeloid and lymphoid lineages.

In region R1, 0.1% to 2.8% of the cells were positive for both CD34 and CD45. It is also noteworthy that 1.1% of the cells were CD34+/CD45- (Figure 2). In region R2, the cell immunophenotypes that were most frequently positive (> 30%) included CD34+/CD45+, CD117+/CD45+, CD13+/CD45+, CD33+/CD45+, CD22+/CD45+ and CD38+/CD45+ (Figures 1 and 3).

In region R3, the cell immunophenotypes that were most frequently positive (> 30%) included CD117+/CD45+, CD13+/CD45+, CD14+/CD45+, CD33+/CD45+, CD38+/CD45+, CD11c+/CD45+ and CD15+/CD45+ (Figures 1 and 3).

In region R4, the most frequent phenotypes were CD117+/CD45+, CD13+/CD45+, CD14+/CD45+, CD33+/CD45+, CD38+/CD45+, CD61+/CD45+, CD11c+/CD45+, CD15+/CD45+ (> 39%) and CD61+/CD45+ (38%) (Figures 1 and 3).

**Figure 4.**
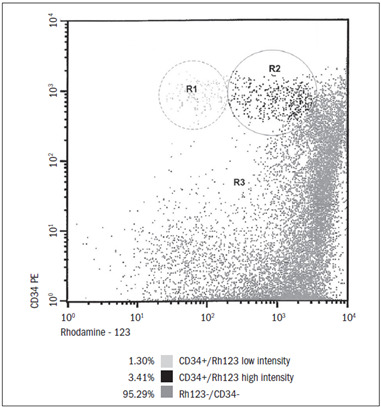
Distribution and percentage of marrow cells according to expression of CD34 and rhodamine uptake. R1: CD34+/low-intensity rhodamine; R2: CD34+/high-intensity rhodamine; R3: CD34-/rhodamine-negative.

After sorting the CD34+ cells, we found a median number of 2.2 x 10^6^ cells, which was equivalent to 4.9% of the total number of cells. With the exception of the patient with severe aplastic anemia, we were able to demonstrate that the higher the number of CD34+ cells was, the earlier the engraftment took place. Our analyses revealed a positive relationship between the number of infused CD34-positive cells and the engraftment day (Table 1). After sorting the CD34 +/CD45+ cells, the proportion of these that coexpressed CD38 was high, ranging from 86.5 to 98.8%, with a median of 94.9% (Table 2). The same cells coexpressed CD117, ranging from 32% to 98.8%, with a median of 82.5%, as shown in Table 3. From analyzing the cells that captured Rh-123 in relation to the total number of mononuclear cells, it was evident that more than 80% of the mononuclear cells expressed high levels of Rh-123 accumulation. By evaluating the CD34+ cells alone, we observed that cells located in the regions with greater differentiation retained Rh-123 more intensively, while the more primitive CD34-positive cells (located in R1) accumulated less Rh-123 (Figure 4).

**Table 1.** Correlation between CD34+ cells and engraftment

Patients	Total CD34+ cells harvested	% CD34+ cells	Engraftment day (D+)
1. BJTA	3.1 x 10^9^	3.9%	18
2. JLPS	16 x 10^9^	13.8%	15
3. MSCP	2.2 x 10^9^	4.0%	*
4. NMSP	4.4 x 10^9^	10.9%	19
5. MLG	2.0 x 10^9^	4.9%	23
6. ESK	2.0 x 10^9^	2.1%	22
7. RRST	5.9 x 10^9^	6.7%	18
8. EP	2.4 x 10^9^	7.5%	16
9. JFRJ	7.0 x 10^9^	2.7%	23
10. LAS	8.0 x 10^9^	2.0%	17
11. NOS	1.1 x 10^9^	6.6%	25
Median	2.0 x 10^9^	4.9%	18

*death before engraftment.

**Table 2.** Expression of CD34/CD38 after sorting of CD34+/CD45- cells

Patients	After sorting of CD34+/CD45/CD38+ cells
1. BJTA	87.6
2. JLPS	98.8
3. MSCP	95.3
4. NMSP	95.2
5. MLG	91.4
6. ESK	95.6
7. RRST	98.8
8. EP	90.9
9. JFRJ	94.6
11. NOS	86.5
Median	94.9

**Table 3.** Expression of CD34/CD117 after sorting of CD34+/CD45- cells

Patients	After sorting of CD34+/CD45/CD117+ cells
1. BJTA	32.0%
2. JLPS	98.8%
3. MSCP	94.8%
4. NMSP	75.1%
5. MLG	35.2%
6. ESK	89.8%
7. RRST	95.6%
8. EP	48.6%
9. JFRJ	97.1%
11. NOS	44.0%
Median	82.5%

## DISCUSSION

Better understanding of hematopoietic stem cell physiology and subpopulation distribution is still required. Flow cytometry has proved to be a useful tool for this, since it is fast and it identifies and quantifies these subpopulations, in their various stages of maturation.^10^

This study was based on characterization of the hematopoietic stem cell subpopulations in regions R1, R2, R3 and R4, which were already known to include cells with positive expression of the antigens listed above.^8^ Our results were similar to what had previously described, especially with regard of simultaneous expression of myeloid and lymphoid antigens in region R2. In this region, the cells were CD19+/CD22+/CD13+/CD33+, thus indicating very little degree of differentiation. This characteristic has been defined as “antigenic infidelity”.^2^^,^^5^^,^^8^^,^^12^

In the subsequent differentiation steps, we were able to define regions of progressive lineage commitment, with cells expressing antigens that were more specific to definite cell lineages, as shown in regions R3 and R4 (Figures 1 and 3).

Region R1 included cells that were CD45-negative. These had a minimum level of differentiation, and most of them proved to be CD34-positive (Figure 2). The expression of CD45 increased progressively, in parallel with the cell maturation, as seen in regions R3 and R4 (Figure 3).

Using a specific combination of anti-B lymphocyte antibodies, we were able to locate B-cells in the regions according to their degree of maturation. Most B-cell precursors remained in region R2, thus coinciding with the region that included most of the CD34-positive cells (Figure 3). The concomitant expression of myeloid antigens in this region supports the idea that these cells still do not have much differentiation and do not possess lineage-specific antigens alone.

The simultaneous expression of CD45, CD45RA and CD38 in lymphoid B-cells is a peculiar pattern. Both CD45 and CD45RA are increasingly expressed when the CD10 expression declines. On the other hand, CD38 follows CD10 expression, until the cells become mature enough and are released into the blood stream. CD38 will be again expressed when these lymphocytes become activated.^6^^,^^13^^,^^14^^,^^15^

With regard to lymphocyte differentiation, CD10 expression is initially regulated by the presence of CD34, as well as CD3 expression. CD10 is little expressed until the immunoglobulin heavy-chain (IgH) genes have been rearranged and the IgH protein reaches the membrane. CD3 (T-cell receptor) is only expressed after the genes coding for this protein have been rearranged. These characteristics constitute evidence for defining the immunophenotype of the less differentiated B-cells as CD34+/CD19+/CD10+ (low intensity), in the presence of rearranged IgH protein. The region with the highest concentration of B-cell precursors, according to the surface antigen pattern, was R2. In this region, we found that 30% of the cells were CD22+ and 18% were CD19+, i.e. proportions similar to what was found by Loken et al.^16^ and Pontvert-Delucq et al.^12^ At present, identification of a common B-cell/myeloid progenitor by means of absence of CXCR4 in the early CD34+/CD19+ population emphasizes the idea of maturation infidelity among the cells from this region.^6^ However, CD34+/CD19-/CD45RA- with low or absent IL-3Ra and TpoR may distinguish common myeloid/monocyte from megakaryocyte/erythroid precursors.^9^

Chabannon et al.^17^ demonstrated that small populations of CD34+/CD7+ cells may correspond to progenitor cells that still lack lineage commitment and can differentiate to granulocytes, when subjected to long-term marrow culturing. Miller et al.^18^ postulated that these CD34+/CD7+ cells constitute an intermediary stage in natural killer (NK) cell differentiation. In our samples, the median proportion of CD7/CD45 in region R1 represented only 0.5% of the total number of cells in this region (Figure 3).

In myelopoiesis, 41% of the CD34-positive cells also expressed CD33 and CD45 in R2. This percentage increased to 58% in R3 and 68% in R4, with median values that were statistically different from the median R1 value (Figure 3). For the same regions, Syrjälä et al.^8^ found 27% (R2), 73% (R3) and 94% (R4).

Myeloid cells with greater differentiation expressed CD13 and CD45 in a similar way to what was observed by Inaba et al.^19^ We found proportions of CD13+/CD45+ of 53% in R2, 54% in R3 and 54% in R4 (Figure 3).

Immature granulocytic precursors show immunophenotypes akin to monocytes and NK cells. CD11c and CD11b are helpful in identifying granulocytic cells of greater maturity, since they are not expressed in the more immature ones. Adding CD15 to the panel also provides more information for characterizing granulocytes with greater differentiation, especially promyelocytes and myeloblasts.^14^^,^^20^ Neutrophils can be distinguished from eosinophils by the positive expression of CD16 in the former and negative expression in the latter.

We evaluated the presence of granulocytic committed cells by analyzing the expression of CD11c, CD15 and CD14 (monocytes). CD11c+/CD45+/CD34+ cells amounted to 37% in R3 and 49% in R4. R1 and R2 showed no significant expression of these antigens, and this was also observed by Syrjälä et al.^8^ and Terstappen et al.^13^ Monocytic cells (CD14+/CD45+) amounted to 34% of the cells in R3 and 55% in R4. This pattern of expression was significantly different from the expression in regions 1 and 2. Syrjälä et al.^8^ found proportions of CD14+/CD45+ cells of 3% in R3 and 30% in R4. Our results may be explained by contamination of marrow samples with cells of greater maturity from the blood stream, at the time of marrow harvesting for donation.

NK cells express CD56 and are negative for CD3. It has been hypothesized that the NK progenitor is related to the most primitive CD34-positive cells. Miller et al.^18^ obtained mature NK cells from long-term marrow culturing. Our data show that NK cells were present in areas where CD34 was also expressed (values between 8% and 13% in regions 2, 3 and 4, Figure 3). We were unable to compare these data with other groups, since no such studies have been reported, as far as we know. However, a common origin for NK cells and lymphoid and myeloid precursors has been now advocated.^21^^,^^22^^,^^23^

Exposure of CD34-/CD133-/CD7-/CD45dim/lin- hematopoietic stem cells from human cord blood to stem cell factor in long-term marrow cultures has been associated with the following: 1) concordant expression of the antigens CD34 and CD133; 2) generation of colony-forming unit (CFU) granulocyte-macrophages, burst-forming unit erythroids and megakaryocytic aggregates; 3) significant extended long-term marrow culture-initiating cell activity; and 4) upregulation of messenger ribonucleic acid (mRNA) signals for myeloperoxidase. At variance with CD34+/lin- cells, CD34-/CD133-/CD7-/CD45dim/lin- hematopoietic stem cells maintained with IL-15 but not with IL-2 or IL-7 have been found to proliferate and differentiate into a homogeneous population of CD7+/CD45(bright)/CD25+/CD44+ lymphoid progenitors with high expression of the T-cell-associated transcription factor GATA-3. Despite harboring non-clonally rearranged TCRg genes, IL-15-primed CD34-/CD133-/CD7-/CD45dim/lin- hematopoietic stem cells have been found not to achieve full maturation, as manifested in their CD3-/TCRab-/gd- phenotype. Conversely, cultures on stromal cells supplemented with IL-15 have been associated with acquisition of NK cells. Collectively, CD34-/CD133-/CD7-/CD45dim/lin- hematopoietic stem cells from human umbilical cord blood have been found to display exquisite sensitivity to IL-15 and differentiation into lymphoid/NK cells.^21^

Long-term marrow culturing of the CD56-/CD34- myeloid-like adherent cell fraction (ACF) from umbilical cord blood, characterized by CD14+ expression and myeloid markers set up with flt3 ligand (FL) and IL-15, have been found to gradually express CD56, reaching high levels of approximately 90% by day 15. FL plus IL15-driven ACF/CD56+ cells have been found to progressively express an NK program, thereby lysing both NK and LAK-sensitive tumor cells and producing high levels of interferon-gamma (IFN-g), granulocyte/macrophage-colony stimulating factor (GM-CSF), tumor necrosis factor alpha (TNF-a) and IL-10, upon stimulation with IL-12 and IL-18. Similar results have been obtained with highly purified CD14+ cells from umbilical cord blood, cultured with FL and IL-15. In contrast, umbilical cord blood/CD34+ cells cultured under the same conditions have shown delayed expression of CD56, with different behavior in that they exhibited NK but not LAK cytotoxicity and produced significantly fewer cytokines. Kinetic studies on the phenotype of umbilical cord blood/ACF or umbilical cord blood/CD14+ cells has shown a rapid decrease in CD14 expression after day 5, reaching levels of zero by day 20. Approximately 60% of the CD56+ derived from umbilical cord blood/ACF or umbilical cord blood/CD14+ cells has been found to coexpress CD14 by day 5.

Taken together, these data support the hypothesis that CD14+ myeloid-like cells have a role in umbilical cord blood as a novel progenitor for lymphoid NK cells.^22^ Furthermore, early-sorted CD34+/CD56+ stem cells in peripheral blood maintained in long-term marrow cultures with IL-2 and stem cell factor have been found to differentiate into two CD56 cells populations: one CD56bright/CD33- population with large granular lymphocyte morphology and cytoplasmatic granzyme-A but lacking a killer inhibitory receptor (thus suggesting that they are immature NK cells); and another CD56dim/CD33+ population with macrophage morphology that proliferates and produces a variety of cytokines upon lipopolysaccharide stimulation, including IL-18, IL-6, monocyte chemoattractant protein and macrophage-derived chemokines, but not IFN-g.^23^ Thus, true differentiation of CD34 cells to NK cells remains to be determined.

Megakaryocytic precursors have shown CD34 and positive HLA-DR and have exhibited platelet-specific antigens as the maturation process comes to completion (CD41, CD42, CD61, CD36 and CD62).^24^ We observed CD61+/CD45+ cells in R3 (26%) and R4 (38%). Likewise, there are no comparable reports regarding megakaryopoiesis immunophenotypic distribution, to the best of our knowledge. A common CD19-/CD34+/IL-3Ralo+/CD45RA-/TpoR+ megakaryocyte-erythrocyte precursor has been recognized.^9^

Some authors advocated^15^^,^^22^^,^^23^ that true stem cells are CD34+/CD45-/CD38-. This phenotype was found in region R1 in our study (Figure 2). Since only a small proportion of cells express CD34, in relation to the whole volume of cells, with consequent difficulty in isolating them and possible contamination with other cell populations, we chose to perform cell sorting, thereby achieving a considerable degree of specificity.

In order to demonstrate the most primitive hematopoietic stem cell, we coupled the quest for CD34-positive cells with an investigation into which of these cells were CD117 and CD38-positive. These very primitive cells are known to be CD34+/CD38-, but the latter antigen becomes evident in progenitors with greater differentiation (90% positivity) until its disappearance from fully differentiated cells.^13^

Escribano et al.^7^ reported that more than 50% of CD34+ cells also expressed CD117, including progenitors committed to erythroid, granulocytic and monocytic lineages, along with small populations of NK and T-cell precursors. Sperling et al.^25^ and Ashman et al.^26^ stated that 70% of CD34-positive marrow cells were also CD117-positive. Recently, CD34+/CD38- cells were reported to be particularly distinguished by: (1) a capability for secondary hematopoietic reconstitution of human cord blood;^27^ (2) a capability, among CD34+/CD38- cells from umbilical cord blood, to divide and proliferate *in vitro* for at least six months without showing any increase in apoptosis or any numerical or structural chromosomal abnormalities;^28^ and (3) a capability to present genes from megakaryopoiesis, in cells obtained from CD34+/CD38^low^ that were cultured in serum-deprived medium supplemented with IL-3, IL-6, stem cell factor and thrombopoietin.^29^ Thus, CD34+/CD38- cells come very close to being true hematopoietic stem cells.

CD117 expression occurs in myeloid and T-lymphoid lineages, and this molecule determines two differently sized groups of CD34-positive cells, with regard to higher or lower intensity of expression. CD34+/CD117+ cells with low expression form a small population that is believed to reconstitute hematopoiesis after marrow ablation.^7^^,^^14^^,^^20^

Among the ten samples analyzed, we found a median CD34 expression of 4.9%, which was similar to published data. CD34/CD38 coexpression occurred in 94.9% of the events and CD34/CD117 coexpression in 82% (Tables 2 and 3).

Regarding CD34/CD117 coexpression, it was shown that CD117 was absent from the CD34-positive cells located in R1. There have been reports stating that true hematopoietic stem cells do not express CD117 on the membrane, but that CD117 m-RNA can be detected.^7^^,^^25^^,^^26^

Rh-123 is one of the substrates of P-glycoprotein (Pgp), and the presence of active Pgp can be shown by the efflux of Rh-123. Rh-123 can also be used to measure the mitochondrial transmembrane potential (energy state) of a cell. It was reasoned that the quantity of hematopoietic progenitors selected using a combination of Rh-123 efflux and phenotypic markers might be superior to the quantity selected by using phenotypic markers alone.^11^ High Rh-123 retention was seen in 80% of the mononuclear cells in the marrow cell population, and this population was related to the more mature cells. After separation of the CD34-positive cells, 95.3% of the CD34-negative cells were associated with high Rh-123 retention. Moreover, out of the 4.7% of all of the CD34 cells that were positive, 3.4% presented high Rh-123 retention and 1.3% presented low retention. It is remarkable that the total quantity of CD34-positive cells obtained by sorting analysis was the same as the sum of CD34-positive cells with high and low Rh-123 retention cells (Figure 4). This finding leads to the conclusion that the low Rh-123/CD34-positive cells were close in quantity to the CD34-positive and CD45-negative cells found in region R1, and they might represent the most primitive hematopoietic cell population. Transplantation of cell populations with high and low Rh-123 retention into irradiated mouse recipients revealed that the robust megakaryocyte differentiation was from low Rh-123 retention cells. Thus, in an animal model, the differentiation capability of these early CD34 hematopoietic progenitor cells was confirmed.^15^

We highlight that identification of CD34+/CD38- cells associated with low Rh-123 retention is currently of high interest in general medicine, in relation to repairing damage in cardiac, orthopedic and several other diseases. Today, hematopoietic stem cells have become a new focus for treating degenerative diseases.^30^ We foresee that our technique for separating hematopoietic stem cells will form a useful strategy for experimental and therapeutic applications requiring human stem cells in large quantity.

## CONCLUSION

CD34+/CD38- and CD34+/CD117- immunophenotypes were found in region R1, subsets of myeloid and lymphoid precursors were seen in region R2 and defined lineage maturations cells were seen in regions R3 and R4.

The average proportion of CD34+ cells was 4.9% and there was a correlation between the total number of these cells and engraftment day.

High Rh-123 retention was found in 80% of mononuclear marrow cells. This population was related to more mature cells. Overall, in the CD34+ cells, the mean low Rh-123 retention rate was 1.3% and the mean high Rh-123 retention rate was 3.4%. The low Rh-123 retention rate in region R1 was close to the proportion of CD34+ cells found in that region, and this may represent the most primitive stem cell population.

The total number of CD34+ cells obtained by sorting analysis was close to the sum of CD34+ cells with high and low Rh-123 retention.

We advocate that true stem cells are CD34+/CD45-/CD38-/low-Rh-123 accumulations.
